# Possible Interruption of Measles Virus Transmission, Uganda, 2006–2009

**DOI:** 10.3201/eid1701.100753

**Published:** 2011-01

**Authors:** Frederick N. Baliraine, Josephine Bwogi, Henry Bukenya, Ronald Seguya, Theopista Kabaliisa, Annet Kisakye, William B. Mbabazi, Sheilagh B. Smit

**Affiliations:** Author affiliations: University of California, San Francisco, California, USA (F.N. Baliraine);; Uganda Virus Research Institute, Entebbe, Uganda (J. Bwogi, H. Bukenya, R. Seguya, T. Kabaliisa);; Uganda National Expanded Program for Immunization, Entebbe (A. Kisakye);; World Health Organization, Kampala, Uganda (A. Kisakye);; Management Sciences for Health/Strengthening Pharmaceutical Systems, Juba, Sudan (W.B. Mbabazi);; National Institute for Communicable Diseases, Johannesburg, South Africa (S.B. Smit)

**Keywords:** viruses, measles, interruption, genotype B3.1, Uganda, dispatch

## Abstract

To determine what measles virus genotype(s) circulated in Uganda after strategic interventions aimed at controlling/eliminating measles, we examined samples obtained during 2006–2009 and found only genotype B3.1, which had not been previously detected. Kenya was the likely source, but other countries cannot be excluded.

In October 2002, Uganda implemented a 5-year (2002–2006) accelerated measles control strategy that began with a vigorous attempt to interrupt all chains of measles transmission by using a 5-day countrywide vaccination campaign. This brisk catch-up campaign was preceded by vaccine potency tests; meticulous planning to ensure political, religious, and tribal leaders’ support; spirited social mobilization; training of health care workers and volunteers; and adequate provision of vaccination and cold chain materials at all vaccination posts, some of which were improvised structures (e.g., tents, schools, or under trees) for easy access. Community education was particularly vital to dispel commonly held myths that vaccinating children against measles or taking children having measles to the hospital (i.e., using foreign medicine) increases the risk for death. About 13.5 million (≈0.5 million above target) children ages 6–168 months were vaccinated, giving a national measles vaccine coverage rate of 104% (*1*). This was followed by keep-up campaigns in 15 high-risk districts for children ages 6–23 months in February 2005 and in April 2005 for all previously unvaccinated children ages 9–59 months. Uganda was virtually measles free in 2003–2005, but outbreaks resurfaced in 2006. Subsequently, nationwide follow-up supplemental measles vaccination campaigns were conducted for children ages 6–59 months during August–November 2006 (*1*) and for children ages 9–47 months in June 2009.

Virologic surveillance before initiating accelerated measles control activities enables genotypes to be cataloged in a country both before and after vaccination campaigns, which together with standard epidemiologic data can help detect imported viruses and evaluate control strategies (*2*). In Uganda, measles virus isolation began in 2000 (*3*). Our study sought to determine the measles virus genotype(s) circulating in Uganda after strategic interventions aimed at controlling/eliminating measles in the country were implemented.

## The Study

**Figure 1:**
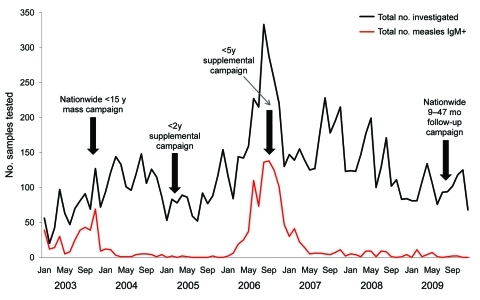
Laboratory-confirmed measles cases in Uganda, 2006‒2009. Data from the accelerated measles control period 2003‒2005 are included for comparison. The surge in measles cases during 2006 was caused by a resumption of measles outbreaks after a 3-year lag period, due to an accumulated number of susceptible persons (*1*).

As part of routine measles case investigations, urine samples and throat swab specimens for virus isolation were collected along with serum samples (0–12 days after rash onset) from patients across Uganda during 2006 through 2009. Infections were confirmed serologically or by virus isolation (*4**,**5*). Of the serum samples tested, 1,053 (15%) of 6,999 were positive for measles immunoglobulin (Ig) M; most were collected during 2006 (Figure 1). Twenty-two isolates (37%) were obtained from 59 samples from patients who had IgM against measles virus; 1 isolate was obtained from a patient who did not have IgM against measles virus; and another isolate was obtained from a patient who did not have serologic testing. Virus isolation was successful only in specimens collected within 5 days of rash onset (Table).

**Table:** Epidemiologic data associated with measles virus isolates analyzed, Uganda, 2006‒2009*

Isolate name	District	Patient age, mo	Measles vaccine doses	Vaccination card seen	Date of onset	Interval, d†	Measles IgM	GenBank accession no.	Identity‡
MVi/Bushenyi.UGA/43.06	Bushenyi	19	1	No	2006 Oct	4	Pos	GU952229	A
MVi/Hoima.UGA/7.09/1	Hoima	36	X	No	2009 Feb	5	Pos	GU952246	B
MVi/Hoima.UGA/7.09/2	Hoima	36	X	No	2009 Feb	2	Neg		B
MVi/Kampala.UGA/26.06/1	Kampala	30	X	No	2006 Jun	1	Pos	GU952239	A
MVi/Kampala.UGA/26.06/2	Kampala	36	X	No	2006 Jun	4	Pos		A
MVi/Kampala.UGA/26.06/3	Kampala	18	0	No	2006 Jun	2	ND	GU952237	C
MVi/Kasese.UGA/7.07/1	Kasese	25	0	Yes	2007 Feb	2	Pos	GU952245	A
MVi/Kasese.UGA/7.07/2	Kasese	240	X	No	2007 Feb	3	Pos		A
MVi/Kitgum.UGA/28.06	Kitgum	60	2	No	2006 Jul	3	Pos	GU952235	A
MVi/Mukono.UGA/26.06	Mukono	96	0	No	2006 Jun	1	Pos	GU952238	D
MVi/Mukono.UGA/29.06	Mukono	276	X	No	2006 Jul	3	Pos	GU952233	E
MVi/Mukono.UGA/47.06	Mukono	48	1	Yes	2006 Nov	1	Pos	GU952243	F
MVi/Wakiso.UGA/26.06	Wakiso	36	2	No	2006 Jun	1	Pos	GU952240	A
MVi/Wakiso.UGA/27.06	Wakiso	96	0	No	2006 Jul	1	Pos	GU952236	C
MVi/Wakiso.UGA/29.06	Wakiso	33	0	Yes	2006 Jul	1	Pos	GU952234	A
MVi/Wakiso.UGA/31.06/1	Wakiso	19	1	No	2006 Jul	1	Pos	GU952232	G
MVi/Wakiso.UGA/31.06/2	Wakiso	25	0	No	2006 Aug	2	Pos	GU952231	A
MVi/Wakiso.UGA/32.06	Wakiso	11	X	No	2006 Aug	1	Pos	GU952230	A
MVi/Wakiso.UGA/41.06	Wakiso	27	0	No	2006 Oct	1	Pos	GU952241	A
MVi/Wakiso.UGA/45.06	Wakiso	120	2	No	2006 Nov	2	Pos	GU952242	H
MVi/Wakiso.UGA/49.06	Wakiso	216	1	No	2006 Dec	2	Pos	GU952244	A

*All were genotype B3.1. IgM, immunoglobulin M; pos, positive; neg, negative; X, unknown; ND, not done. †Time between rash onset and sample collection. ‡Nucleotide sequences sharing the same letter are identical.

**Figure 2:**
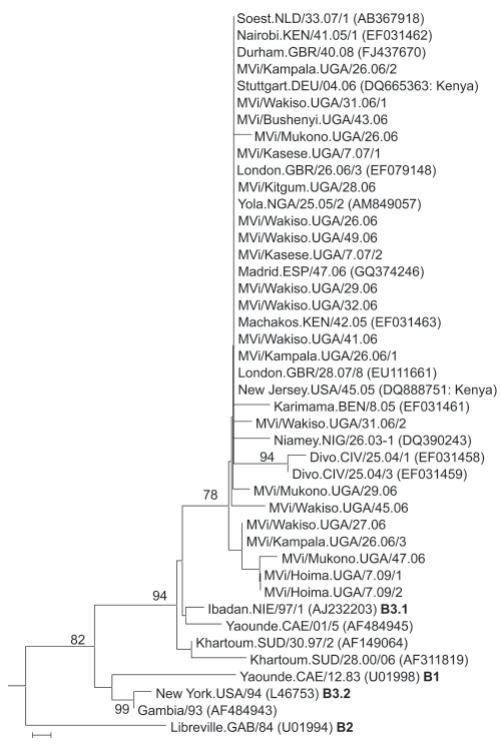
Phylogenetic analysis of the relationship between sequences of 21 Ugandan measles virus isolates obtained during 2006–2009 and 22 other recently described clade B nucleoprotein (N) gene sequences, including the World Health Organization reference strains for the B clade (*13*). **Boldface** indicates different genotypes. Analyses are based on sequences of the 450 nt encoding the COOH-terminal 150 nt of the N gene. The unrooted neighbor-joining consensus tree was generated by bootstrap analysis of 500 replicates by using MEGA4 software (www.megasoftware.net). Bootstrap percentages are shown when >75%. Only names of the isolates from Uganda (UGA) start with “MVi,” and all comparison strains have their GenBank accession numbers indicated in parentheses. Genotypes of the World Health Organization reference sequences are indicated after the accession number. Comparison sequences were from viruses isolated in Benin (BEN), Cameroon (CAE), Côte d’Ivoire (CIV), Gabon (GAB), the Gambia, Germany (DEU), Great Britain (GBR), Kenya (KEN), Niger (NIG), Nigeria (NIE/NGA), Spain (ESP), Sudan (SUD), the Netherlands (NLD), and the United States (USA). Sequences from Uganda were most closely related to the B3.1 viruses of the September–December 2005 measles outbreak in Kenya (*8*). Scale bar indicates nucleotide substitutions per site.

All isolates belonged to genotype B3.1 (Figure 2), which had not been previously detected in Uganda (*3*). Twelve (57%) of 21 sequences obtained were identical (Table) and also identical to isolates from the 2005 measles outbreak in Kenya (Figure 2). However, 9 (43%) of 21 showed neither 100% similarity with the other 12 Ugandan isolates (Table) nor with any other isolate available in GenBank.

Since the inception of Uganda’s 2002–2006 accelerated measles control strategic plan, the number of measles cases in the country has declined dramatically (Figure 1). After the 2003 campaign, virtually no cases of measles occurred in Uganda for 3 years, until the outbreaks in 2006 (*1*). Our data show that 819 (78%) of 1,053 of the serologically confirmed measles cases for the 4-year surveillance period occurred during the 2006 outbreaks (Figure 1), confirming that the strategic interventions quickly subdued the 2006 transmission cycles. Moreover, the pattern of measles genotypes detected from 2000–2009 (2006–2009 reported in this study) suggests that transmission of the previously endemic genotype D10 in Uganda (*3*) has been interrupted and replaced by genotype B3.1. However, since molecular surveillance in Uganda only began 10 years ago (*3*), we cannot ascertain which genotypes were circulating in the country before 2000. By 2001, B3.1 was geographically restricted to the western and central African countries of Ghana, Nigeria, Cameroon, and Sudan (*6**,**7*); however, by the end of 2005, it had spread to Kenya, supposedly from Nigeria, where it caused a massive epidemic that was linked to subsequent infections in Europe and the Americas (*8*).

The fact that most isolates from Uganda were identical to those previously identified in neighboring Kenya indicates that Kenya was the most likely immediate source of the B3.1 viruses presently circulating in Uganda. However, the contribution of other African countries cannot be excluded, because molecular surveillance is still largely lacking in Africa (*5*).

Only 14 (23%) of 61 patients with laboratory-confirmed measles (whose specimens were screened for measles virus isolates) had been vaccinated, demonstrating a gap in vaccination coverage and possible vaccination failure in some cases and the need for timely catch-up vaccination campaigns even in the low-risk districts. In Uganda, routine childhood vaccination against measles began in 1983, but vaccine coverage was initially too low to interrupt indigenous transmission. The situation was not helped by the rampant poverty, limited health-care infrastructure, internal and external conflicts that have rocked the Great Lakes Region during the past 3 decades, high vaccination drop-out rates, and a high birth rate (second highest in the world, after Niger [*9*]). These conditions are typical in countries where vaccine-preventable infections remain a major problem (*10*). Nevertheless, education of Ugandan health workers, politicians, and development partners has been ongoing. This strategy has aroused strong interest and support for disease surveillance and control activities from the political leadership, resulting in the creation of a special budget-line for surveillance (*11*). Since 2003, routine vaccination has been strengthened by extending primary health care grants to all districts, and the implementation of the “Reaching Every District” strategy (*12*), among other interventions. Consequently, Uganda has recorded a tremendous rise in vaccination coverage, from 64% in 1997 to >85% by 2007 (*1*). This aggressive vaccination effort has been instrumental in interrupting indigenous measles strain transmission in the country.

All virus isolates from Uganda during 2000–2002, before accelerated measles control, belonged to genotype D10 (*3*). At that time, viruses isolated in countries bordering Uganda, such as Kenya, Sudan, and the Democratic Republic of the Congo, belonged to genotypes D4, B3, and B2 (*13*). However, without enhanced regional surveillance, should genotype D10 be isolated again, it would be difficult to determine whether it had truly been interrupted, without knowing if D10 was presently circulating in other African countries, which could serve as bases from which to reintroduce it into Uganda.

## Conclusions

Our results show that, even under difficult circumstances (e.g., poverty), optimal resource allocation and mobilization of political will can interrupt vaccine-preventable diseases in Africa. These data provide molecular evidence that Uganda’s 2002–2006 vaccination strategy was successful in interrupting indigenous measles transmission, but immunity gaps in the population allowed the establishment of an imported virus that was previously confined to western and central Africa. If national immunization programs across the region synchronized their vaccination strategies to eliminate sources of reintroduction, measles could be quickly eliminated from the entire continent. Vaccination success stories have already been noted in several African countries with routine coverage >80% (*14**,**15*). Therefore, continued education and cooperation are needed between countries, national policy makers, health care workers, and local communities throughout the continent to win the fight against measles.
